# Leisure involvement, social inclusion, and happiness in Türkiye: a mediational analysis

**DOI:** 10.3389/fpubh.2025.1696910

**Published:** 2025-11-26

**Authors:** Davut Budak, Elvan Deniz Yumuk

**Affiliations:** 1 Sports Sciences Faculty, Atatürk University, Erzurum, Türkiye; 2 Sports Sciences Faculty, Alanya Alaaddin Keykubat University, Antalya, Türkiye

**Keywords:** leisure involvement, social inclusion, happiness, mediation effect, Türkiye

## Abstract

**Background:**

Engagement in meaningful leisure activities is increasingly recognized as an essential factor for promoting psychological wellbeing and happiness. However, the mechanisms underlying this relationship remain underexplored. This study examines the mediating effect of social inclusion in the relationship between leisure involvement and happiness among Turkish adults.

**Methods:**

The study employed a cross-sectional survey design and included 595 adult participants who were recruited from seven regions of Türkiye. Data were collected through self-administered questionnaires comprising the Leisure Involvement Scale (LIS), the Oxford Happiness Questionnaire–Short Form (OHQ-SF), and the Social Inclusion Scale (SIS). Mediation analysis using Hayes' PROCESS macro (Model 4) was conducted to assess the mediating role of social inclusion in the relationship between leisure involvement and happiness.

**Results:**

Leisure involvement was positively associated with both social inclusion (*r* = 0.37, *p* < 0.001) and happiness (*r* = 0.37, *p* < 0.001). Social inclusion was also strongly correlated with happiness (*r* = 0.50, *p* < 0.001). Mediation analysis indicated that social inclusion partially mediated the correlation between leisure involvement and happiness. The total effect of leisure involvement on happiness was β = 0.37, with a direct effect of β = 0.21 and an indirect effect through social inclusion of β = 0.16.

**Conclusion:**

Social inclusion is a significant psychological mechanism that enhances the positive impact of leisure involvement on happiness. Promoting socially inclusive leisure opportunities may serve as an effective public health strategy for improving subjective wellbeing across diverse community settings. These findings suggest that community programs, urban planning, and public policies should prioritize accessible and inclusive leisure activities to strengthen social connections and enhance happiness, particularly among marginalized groups.

## 1 Introduction

In contemporary society, participation in positive and meaningful leisure activities is increasingly recognized as vital for overall wellbeing. In particular, regular physical activity during leisure has been shown to offer a range of benefits, including improved physiological and psychological health (1–3). Leisure activities play a crucial role in helping individuals cope with daily life challenges while maintaining their health and overall wellbeing (4). Previous studies have consistently found that participation in leisure activities yield positive outcomes for mental health and subjective wellbeing (5–7). Within this framework, engaging in leisure activities is conceptualized as leisure involvement, which refers to the strength or extent of cognitive, affective, and behavioral connections between an individual and a leisure activity (8).

Leisure involvement is also used to explain individuals' personal choices for leisure activities (9). The three main components of leisure involvement, namely attraction, centrality and self-expression, are widely discussed in the literature. Attraction in particular is closely linked to the emotional dimension of an individual's attitude (10). Centrality, on the other hand, is related to conative aspect whereas self-expression is considered to be connected to the cognitive aspect. When all these pillars of an attitude of engaging in a leisure activity are considered, the role of this engagement in individuals' psychological health must be taken into account as well. Although leisure involvement is believed to impact outcomes such as life satisfaction (11), there remains a notable gap in the literature regarding the relationships among leisure involvement, social inclusion, and happiness. However, few studies have specifically examined how social inclusion may mediate the relationship between leisure involvement and happiness, particularly in the context of Turkish adults. This study therefore aims to examine the mediating role of social inclusion in the relationship between leisure involvement and happiness among Turkish adults.

### 1.1 . Background literature and hypotheses

#### 1.1.1 Leisure involvement and its role in psychological health

Leisure involvement encompasses the perceived significance of a recreational activity as well as its symbolic representation of individual identity and its integration into an individual's lifestyle and social relationships (12). Existing literature indicates that participation in leisure activities exerts a positive influence on the perception of associated benefits (13). Scholars have highlighted the positive association between leisure engagement and psychological health, noting that those who participate actively in meaningful leisure activities report higher levels of life satisfaction and emotional stability (14, 15). Research indicates that even small-scale sources of satisfaction can exert a meaningful influence on overall life experiences (16). When individuals derive enjoyment and fulfillment from leisure activities, they are more likely to satisfy their core psychological needs through active engagement, which in turn enhances their overall wellbeing (17). Consequently, the degree of leisure involvement and the fulfillment of psychological needs together contribute to an individual's overall happiness.

### 1.2 The relationship between leisure activities and happiness

Considering the broader perspective of wellbeing, it is useful to examine the philosophical notion of the ultimate good, or summum bonum. The concept of summum bonum, the highest or ultimate good, has been sought since ancient times (18). In contemporary society, this pursuit of ultimate good often translates into seeking happiness through various means, such as higher income, prestigious careers, favorable living environments, or material possessions (19). Recent studies indicate that happiness has a U-Shape across ages (20, 21) meaning that whereas the young and the older adults are more prone to happiness, individuals in their middle ages may suffer from insufficiency of happiness. Waldinger and Schulz (22) emphasize that the concept of a good life, that is happiness, is complex, encompassing dilemmas such as joy and challenge, love and pain. Happiness does not simply occur but unfolds through experiences. Deriving from this point of view, leisure activities can encompass these elements, underscoring their importance in wellbeing through experience. From this perspective, leisure activities can provide opportunities to experience joy, challenge, and fulfillment, linking the concept of leisure engagement to the pursuit of ultimate wellbeing.

Liu and Da (23) declared that happiness is influenced by feelings arising from leisure activities, such as relaxation, tranquility, achievement, autonomy, relatedness, and interest. Similarly, Liu et al. (24) reported that leisure satisfaction contributes to happiness by enhancing individuals' sense of connection and fulfillment. Leisure participation not only improves wellbeing but also facilitates social inclusion, as engaging in recreational activities fosters belonging and shared identity (25–28). Further academic inquiry is essential to investigate the operational mechanisms of social inclusion in mediating the relationship between leisure engagement and happiness, particularly within the context of collectivist cultures such as Türkiye. The findings of aforementioned studies raise a new question: whether social inclusion mediates the relationship between leisure involvement and happiness.

### 1.3 Social inclusion as a mediator between leisure involvement and happiness

Social inclusion involves integrating marginalized or excluded individuals into mainstream society (29). It is a process by which individuals gain opportunities to participate in social life, fulfill their roles, and achieve respect and recognition (30). Participation in leisure activities contributes to social integration and plays a pivotal role in supporting mental wellbeing, developmental outcomes, and cognitive enhancement (6). Kraus (31) describes the social values of recreation, where individuals develop healthy socialization by adapting to group norms and roles. Social inclusion is shaped by various demographics, including socioeconomic status, language, ethnicity, religion, residence, gender, sexual orientation, age, and employment (32).

Empirical and theoretical evidence indicates that social inclusion serves as a central mediator in the relationship between leisure involvement and wellbeing, as engagement in leisure activities not only provides intrinsic enjoyment but also cultivates a sense of belonging and interpersonal connectedness (33, 34). Individuals experiencing greater social inclusion tend to report higher levels of happiness and life satisfaction (35, 36), especially in collectivist cultures where social bonds are central to psychological wellbeing (37). This underscores the vital role of leisure in nurturing both individual and communal wellbeing.

From a theoretical perspective, social inclusion encompasses a sense of acceptance, belonging, and connection within social environments. It functions both as an outcome and facilitator of leisure participation. Integrative reviews indicate that community-based recreation, physical, creative, or social, enhances social inclusion by fostering meaningful interpersonal connections (38). The DRAMMA framework (Detachment, Recovery, Autonomy, Mastery, Meaning, Affiliation) underscores that affiliation in leisure supports subjective wellbeing by fulfilling the need for belongingness (39). Involvement in group-based or community leisure activities also enhances social capital, contributing to increased happiness (40).

Building on prior empirical evidence, increased engagement in leisure activities has been shown to enhance social integration, strengthen social networks, and foster a heightened sense of belonging within the community (41). The support individuals receive from their social environment, in turn, increases their tendency to engage in leisure pursuits (42). From the perspective of self-determination theory, participation in leisure activities is known to facilitate the satisfaction of basic psychological needs such as autonomy, competence, and relatedness (43). The fulfillment of these psychological needs subsequently contributes to enhanced wellbeing (44) and happiness (45). Consequently, involvement in leisure activities comes across as a strong predictor of happiness (46). Especially, structured leisure activities are particularly recognized as significant contributors to social inclusion and overall wellbeing (47). In addition, engaging in socially inclusive activities enables individuals to maintain supportive relationships, which can buffer the effects of stress and adversity (48); due to the fact that leisure involvement serves as a prompt for social inclusion (49). Empirical evidence suggests a positive correlation between engagement in meaningful social interaction and the relationship between leisure activities and overall happiness. In essence, the degree to which individuals participate in fulfilling social experiences may serve as a significant mediator, strengthening the link between leisure involvement and happiness. Further research is warranted to explore the underlying mechanisms through which social interaction enhances the positive impact of leisure pursuits on an individual's sense of happiness and life satisfaction. Therefore, the following hypotheses were developed to evaluate the mediation model.

*H1:* Leisure involvement is positively correlated with social inclusion.

*H2:* Leisure involvement is positively correlated with happiness.

*H3:* Social inclusion is positively correlated with happiness.

*H4:* Social inclusion plays a mediating role between leisure involvement and happiness.

**Figure 1:**
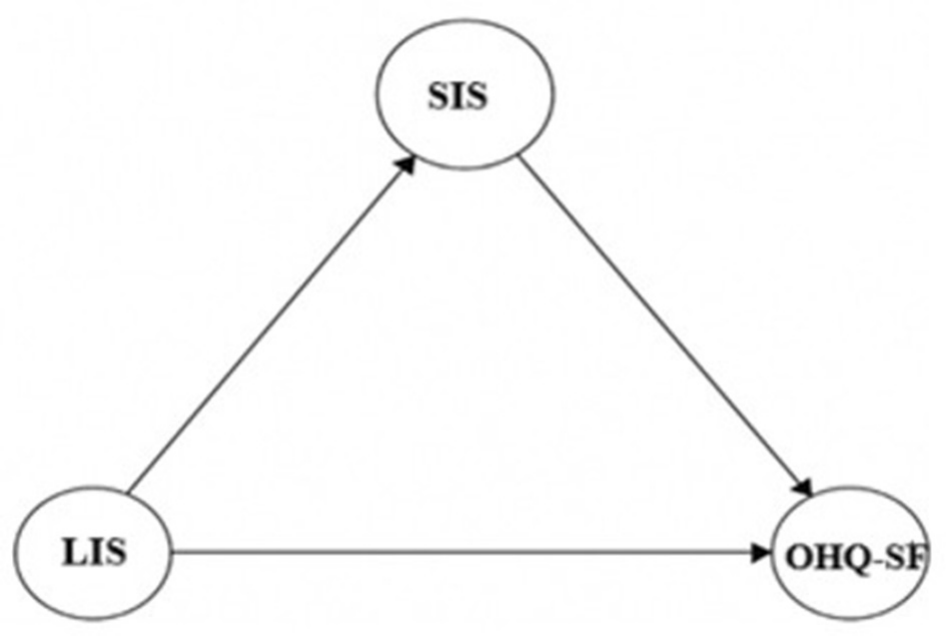
The mediation model.

Based on the hypotheses, the following model is proposed and depicted in Figure 1.

## 2 Methods

### 2.1 Sample and procedure

The sample consisted of 595 voluntary adult participants from Türkiye, including 290 women (48.7%) and 305 men (51.3%)., The participants, ages ranging from 18 to 80, were recruited through convenience sampling. As this study employed convenience sampling, participants were selected based on accessibility. This approach may limit sample representativeness and, consequently, the generalizability of results. The findings should therefore be interpreted with caution and considered primarily within the context of the sampled group. The male participants had a mean age (M) of 32.76 years (SD = 13.12) whereas the females had a mean age of 32.96 (SD=12.96). In the current study, the participants were asked about their marital status (single 64.0% and married 36.0%), education level (high school or lower 12.1% and university or higher 87.9%), income level (low 17.0%, medium 72.6%, and high 10.4%), frequency of physical activity (rarely 32.6%, occasionally 37.8%, and regularly 29.6%), physical activity environment (outdoors 64.4% and indoors 35.6) (Table 1).

**Table 1:** Demographic information.

**Variables**		***N***	**%**
Gender	Male	305	51.3
	Female	290	48.7
Marital status	Single	381	64.0
	Married	214	36.0
Educational level	High school or lower	72	12.1
	University or higher	523	87.9
Income level	Low	101	17.0
	Medium	432	72.6
	High	62	10.4
Frequency of physical activity	Rarely	194	32.6
	Occasionally (1–2 times/week)	225	37.8
	Regularly (≥3 times/week)	176	29.6
Physical activity environment	Outdoors (e.g., park, garden)	383	64.4
	Indoors (e.g., gym, fitness center)	212	35.6

Specifically, participants were recruited from WhatsApp groups and Facebook pages related to recreational clubs, sports activities, community hobby groups, and university student organizations. Participants were invited to take part through posts and messages shared within these groups, and eligibility was confirmed through screening questions at the beginning of the survey. The data was collected throughout August 2023 and January 2024. The objectives of the research and the scientific content were provided to the participants through a comprehensive informed consent form. The anonymity of participants was protected. The inclusion criteria of the study were participants' being older than 18 years of age, participating in leisure activities indoors or outdoors and committed to a regular activity participation. To ensure integrity, incomplete survey forms, and the forms which do not meet the eligibility criteria such as containing substantial missing responses or falling outside the required age range were excluded from the analysis. Therefore, out of 602 survey forms, 595 survey forms were obtained in the final version to be analyzed.

### 2.2 Measures

The data for this study were collected using a structured instrument consisting of two main sections. The first section focused on participants' demographic characteristics as well as their leisure activity participation and preferences. Leisure engagement was assessed through a researcher-developed questionnaire, in which participants reported the frequency of their involvement in leisure activities over the past week (categorized as rarely, occasionally, or regularly). The second section included standardized measurement scales detailed below.

#### 2.2.1 Leisure involvement scale

LIS was developed by Kyle et al. (50) and adapted into Turkish by Gürbüz et al. (51). The Turkish version contains 15 items (e.g., is very important to me) across five subscales: attraction (3 items), significance (3 items), social bonding (3 items), identification (3 items), and expression (3 items). Items are rated on a 5-point Likert scale (1 = strongly disagree, 5 = strongly agree). Factor loadings for the Turkish version ranged from 0.41 to 0.81. In the present study, Cronbach's alpha was calculated as 0.92.

#### 2.2.2 Oxford happiness questionnaire—Short form

OHQ-SF, a widely used measure of subjective wellbeing, consists of seven items (e.g., I feel that life is rewarding), with items 1 and 7 reverse-scored. OHQ-SF was originally developed by Hills and Argyle (52) and later adapted into Turkish by Dogan and Çötok (53). Items are rated on a 5-point Likert scale from 1 (strongly disagree) to 5 (strongly agree). The scale measures general happiness and wellbeing. In this study, Cronbach's alpha was calculated as 0.80.

#### 2.2.3 Social inclusion scale

Originally developed by Secker et al. (54) and adapted into Turkish by Ilgaz, Akgöz, and Gözüm (55), SIS contains 18 items (e.g., I have felt I am playing a useful part in society). Items are rated on a 4-point Likert scale ranging from 1 (not at all) to 4 (yes definitely) and three subscales. Factor loadings for the Turkish version of SIS were 0.40–0.79, explaining 55.14% of variance. Item-total correlations ranged from 0.28 to 0.70, and the content validity index was 0.97. Cronbach's alpha in this study was calculated as 0.89.

### 2.3 Data analysis

In the current study, the influence of leisure involvement on happiness mediated by social inclusion was investigated through the path analysis. All statistical analyses were conducted using SPSS 26.0. Data screening involved checks for missing values and outliers. Skewness and kurtosis values were examined to assess the normality of the data. Values within the range of ±2 are generally considered acceptable for normal univariate distribution in social sciences (56). Mahalanobis distance analysis detected multivariate outliers, and scatterplots confirmed linearity and homoscedasticity. No significant bivariate outliers were found. Descriptive statistics and Pearson correlation coefficients were computed. Hypotheses were tested via Hayes' PROCESS macro (Model 4) for mediation, with 5,000 bootstrapped samples and 95% confidence intervals (57).

A priori power analysis was conducted using G^*^Power 3.1.9.7 to determine the minimum required sample size. Assuming a medium effect size (*f*^2^ = 0.15), power of 0.95, and alpha level of 0.05, the required sample size was calculated to be 138 (58). The actual sample size (*N* = 595) exceeded this threshold, providing adequate statistical power to detect mediation effects. A cross-sectional, correlational survey design was utilized to examine the mediating effect of social inclusion on the correlation between leisure involvement and happiness.

## 3 Results

### 3.1 Descriptive statistics

Descriptive statistics and the correlation matrix for the variables in the model—leisure involvement (independent variable), happiness (dependent variable) and social inclusion (mediating variable)- were assessed via correlation analysis. The related results are presented in Table 2.

**Table 2:** Descriptive statistics and correlation matrix of all variables.

**Scales**	**M ±SD**	**1**	**2**	**3**
1. Leisure involvement scale	3.56 **±** 0.79	**–**		
2. Social inclusion scale	3.32 **±** 0.42	0.37^**^	**–**	
3.The Oxford happiness questionnaire-short form	3.63 **±** 0.67	0.37^**^	0.50^**^	**–**

*N* = 595. ^**^p < 0.01.

The conducted analysis revealed that the mean scores for all three scales indicate moderate to high levels of the respective constructs among participants, with leisure involvement (M = 3.56, SD = 0.79), social inclusion (M = 3.32, SD = 0.42), and happiness (M = 3.63, SD = 0.67). Skewness and Kurtosis values for each scale ranged between −0.51 and −0.19 for Skewness, and −0.49, and 0.36 for kurtosis, indicating acceptable univariate normality (56). Internal consistency was satisfactory across all scales, with Cronbach's Alpha values of 0.93 for Leisure Involvement Scale, 0.80 for Social Inclusion Scale, and 0.76 for The Oxford Happiness Questionnaire-Short Form, exceeding the commonly accepted threshold of 0.70 (59). Furthermore, the Average Variance Extracted (AVE) and Composite Reliability (CR) values were computed using standardized factor loadings obtained from confirmatory factor analysis, and both supported the convergent validity of the measures, with AVE values above 0.50 and CR values above 0.80 for all constructs (60). Specifically, LIS was moderately correlated with both SIS (*r* = 0.37, *p* < 0.001) and OHQ-SF (*r* = 0.37, *p* < 0.001), while SIS demonstrated a stronger correlation with OHQ-SF (*r* = 0.50, *p* < 0.001). These findings are consistent with theoretical expectations suggesting that perceptions of external and internal support mechanisms are linked to subjective wellbeing outcomes. The strength and significance of these relationships not only suggest theoretical relevance but also provide empirical justification for conducting a mediation analysis. Given the intermediate role SIS plays between LIS and OHQ-SF, further testing via structural modeling is warranted.

The mediation analysis was conducted using Hayes' PROCESS macro (Model 4) with 5,000 bias-corrected bootstrap samples to estimate indirect effects. The results showed that LIS positively and significantly predicted SIS [β = 0.37, SE = 0.02, 95% CI [0.33, 0.41]], suggesting that individuals who have higher levels of leisure involvement also report higher levels of social inclusion. In turn, SIS significantly predicted OHQ-SF [β = 0.42, SE = 0.04, 95% CI [0.53, 0.75]], indicating that greater social inclusion is associated with higher levels of happiness. Importantly, the direct effect of LIS on OHQ-SF remained significant after including SIS as a mediator [β = 0.21, SE = 0.03, 95% CI [0.11, 0.24]], supporting a partial mediation model. The indirect effect from LIS to OHQ-SF via SIS was statistically significant [β = 0.16, BootSE = 0.02, 95% CI [0.11, 0.20]], confirming that SIS acts as a mediating mechanism in the relationship between leisure involvement and happiness (Table 3). This suggests that leisure involvement contributes to happiness not only directly but also indirectly through its impact on social inclusion. The conceptual mediation model illustrates the hypothesized and empirically supported indirect pathway from LIS to OHQ-SF via SIS (Figure 1). The model reflects both the direct effect of LIS on OHQ-SF and an indirect pathway through SIS, providing evidence for partial mediation. Meanwhile, Table 4 illustrates the proportion of variance explained in the mediation model, alongside corresponding effect sizes, providing insight into the strength and practical significance of the observed relationships. Specifically, the combined model (LIS & SIS) explained 29% of the variance in OHQ-SF (*R*^2^ = 0.29, *f*^2^ = 0.41), while LIS alone explained 14% of the variance in SIS (*R*^2^ = 0.14, *f*^2^ = 0.16). According to Cohen's (61) benchmarks, Model 1 demonstrated a medium effect (*f*^2^ = 0.16 > 0.15) and Model 2 demonstrated a large effect (*f*^2^ = 0.41 > 0.35), indicating meaningful predictive relationships. These results offer substantiation for the study's hypotheses, in particular, indicating that they were empirically supported (Tables 3, 4).

**Table 3:** Mediating effect of social inclusion on the relationship between leisure involvement and happiness.

**Effects**	**Standardized**			
	β	**standard error**	**LLCI**	**ULCI**
LIS → SIS (a)	0.3704^**^	0.0210	0.1624	0.2448
SIS → OHQ-SF (b)	0.4218^**^	0.0564	0.5267	0.7484
LIS → OHQ-SF (c')	0.2108^**^	0.0310	0.1143	0.2361
LIS → SIS → OHQ-SF (c)	0.1562	0.0221	0.1158	0.2017

*N* = 595, ^**^p < 0.01. LIS, Leisure involvement scale; SIS, Social inclusion scale; OHQ-SF, Oxford happiness scale-short form.

**Table 4:** Model summary statistics.

**Models**	***R* ^2^**	***F***	**df1**	**df2**	***p***	***f* ^2^**	**Effect size**
Model 1: LIS → SIS	0.1372	94.2896	1	593	< 0.001^**^	0.1590	Medium
Model 2: LIS + SIS → OHQ-SF	0.2882	119.8613	2	592	< 0.001^**^	0.4049	Large

*N* = 595. ^**^p < 0.01. LIS, Leisure involvement scale; SIS, Social inclusion scale; OHQ-SF, Oxford happiness scale-short form; β, unstandardized regression coefficient; LLCI/ULCI, Lower/upper limits of 95% bias-corrected bootstrap confidence intervals based on 5,000 samples. Effect sizes calculated using Cohen's (61) formula *f*^2^ = *R*^2^/(1-*R*^2^), where *f*^2^ ≥ 0.1500 indicates medium effect and *f*^2^ ≥ 0.3500 indicates large effect.

## 4 Discussion

Although numerous studies have examined leisure activities, they have predominantly addressed leisure involvement (62), happiness (63), and social inclusion (64) within different constructs, often overlooking their potential interrelations and the broader socio-cultural context in which leisure activities occur. Therefore, the current study explored the mediating role of social inclusion (SIS) in the correlation between leisure involvement (LIS) and happiness (OHQ-SF). With this aim in mind, the first hypothesis indicating that leisure involvement is positively correlated with social inclusion was confirmed. This finding highlights the dual nature of leisure as both personal and social. Policymakers and urban planners should prioritize such initiatives to enhance social cohesion and happiness, particularly in urban settings (27). Moreover, participation in leisure activities is recognized as a domain for diverse community engagement and interaction across socioeconomic strata paving the way to a social cohesion and social capital (65). Therefore, designing inclusive leisure programs that encourage engagement, and belonging could serve as effective strategies to promote community interaction. In particular, such initiatives could be instrumental in strengthening social inclusion and social cohesion when the increasingly urbanized lifestyles of individuals are in question.

The findings of the current study supported the second hypothesis, which posited a positive correlation between leisure involvement and happiness. Consistent with previous research, leisure involvement emerged as a significant predictor of happiness across diverse populations including older adults, adolescents and university students (63, 66, 67). The direct effect of leisure involvement on happiness suggests that leisure also offers intrinsic psychological benefits, possibly through experiences such as flow and autonomy (14, 24). Similarly, Matsumoto et al. (68) found that leisure satisfaction mediates the relationship between affective and cognitive leisure involvement and subjective happiness, further supporting the indirect pathways through which leisure activities contribute to wellbeing. These insights reinforce the significance of promoting quality leisure experiences as a pathway to overall happiness in different social contexts.

Research indicates that active leisure participation is linked to enhanced subjective wellbeing (69). The third hypothesis, predicting a positive correlation between social inclusion and happiness, was confirmed. Social inclusion, by fostering belonging and providing social support, appears to be a significant contributor to happiness (70). Hossen and Salleh (71) developed a model stating that some key variables, including social inclusion, are a path to psychological wellbeing. The benefits of social inclusion include identity formation and community participation, both of which are crucial to the wellbeing of individuals. The results highlight the crucial role of social inclusion in promoting happiness. This suggests that individuals who feel a sense of belonging and receive support through social connections are more likely to experience even greater happiness. Thus, considering social aspects when examining psychological acquisitions is significant because social inclusion not only enhances happiness but also contributes to active participation in community life.

Correlational analyses revealed significant positive correlations among these variables, supporting prior findings that involvement in leisure activities fosters social inclusion and enhances wellbeing (1, 72, 73). Importantly, the mediation analysis confirmed social inclusion as a crucial psychological mechanism that partly explains how leisure involvement contributes to happiness, confirming the fourth hypothesis in the current study. The positive effect of leisure involvement on happiness can be partly attributed to the mediating role of social inclusion. This emphasizes the importance of social connections within leisure activities. Moreover, this finding aligns with the DRAMMA model, which emphasizes the roles of affiliation and meaning in promoting subjective wellbeing through leisure (39). The results highlight the broader psychosocial benefits of leisure activities such as providing more than only individual enjoyment and encompassing sentiments of belonging and social integration. Moreover, extensive literature supports that social inclusion enhances wellbeing and mitigates the detrimental effects of exclusion (74–76). This points out the significance of socially inclusive environments in terms of buffering against the negative effects of exclusion and promoting happiness. The role of social inclusion in leisure and wellbeing appears universal, as seen in cross-cultural studies (77). Echoing our findings, Wang et.al. (69) found that being socially integrated has a mediating role in the correlation of leisure patterns and subjective wellbeing which is a recognized proxy for happiness. Moreover, participating in regular social activities is recognized to contribute to wellbeing and social inclusion (78). The conjunction of these findings underscores the formative role of social interaction in happiness and overall psychological health, emphasizing the significance of designing recreational programs that can promote social inclusion and integrity across diverse populations on the grounds that social inclusion through leisure may be a foundational element of happiness.

## 5 Conclusion

The present research aimed to analyze the mediating effect of social inclusion in the relationship between leisure involvement and happiness. Correlational analyses revealed significant positive correlations among leisure involvement, social inclusion and happiness. Moreover, these findings of the current study suggest that leisure involvement in leisure activities can play a crucial role in increasing both social inclusion and happiness. This study also demonstrates that leisure involvement positively influences happiness both directly and indirectly through social inclusion. Social inclusion serves as a key mediator, showing that inclusive leisure participation can enhance happiness. The moderate to large effect sizes observed underscore the practical importance of these findings for public health.

Promoting inclusive leisure opportunities may offer an accessible, culturally adaptable strategy to improve happiness and social cohesion, especially in increasingly urbanized and fragmented populations where participants live a more individualistic way of life. Also, when the developing trend in “online life” is considered, the importance of leisure activities held in leisure with a group becomes prominent since the fear of missing out actually makes individuals miss out on life. Research underlines the significance of participating actively in leisure programs. Early exposure to leisure activities can significantly facilitate leisure awareness during formative years. Related skills can foster a holistic approach to personal development resulting in physical and mental wellbeing. A comprehensive approach to leisure involvement should be incorporated into programs to be generated. Policymakers and community planners should prioritize accessible, socially engaging leisure programs from a young age to support mental health and wellbeing for achieving overall happiness in all stages of life.

## 6 Limitations

Although the study presents several strengths, we acknowledge certain limitations that should be considered. The cross-sectional design of the current study restricts causal inference; therefore, future longitudinal or experimental research is needed to establish stronger evidence. Exclusive use of self-report measures may introduce bias; thus, subsequent studies should incorporate objective assessments or multi-method approaches. The sampling was limited to a specific geographic area with relatively homogeneous socio-cultural characteristics, potentially limiting the generalizability of the findings to more diverse populations. Demographic moderators were not extensively analyzed and merit exploration in future research. Finally, only cognitive aspects of leisure involvement were assessed; future studies should also examine the behavioral and emotional dimensions to provide a more comprehensive understanding of leisure engagement.

## Data Availability

The raw data supporting the conclusions of this article will be made available by the authors, without undue reservation.
